# A State of Art Review: Volatile Organic Compounds and Periodontitis

**DOI:** 10.31661/gmj.v13iSP1.3730

**Published:** 2024-12-31

**Authors:** Mohammad Ghasemirad, Omid Tavakol, Soroush Etesami, Kiana Nikeghbal, Dorsa Nikeghbal, Fatemeh Abedi Diznab, Reza Mahmoudi Anzabi

**Affiliations:** ^1^ Department of Periodontics, Faculty of Dentistry, Rafsanjan University of Medical Sciences, Rafsanjan, Iran; ^2^ Department of Prosthodontist, Faculty of Dentistry, Shiraz Branch, Islamic Azad University, Shiraz, Iran; ^3^ Department of Periodontics, School of Dentistry, Ahvaz Jundishapur University of Medical Sciences, Ahvaz, Iran; ^4^ Department of Oral and Maxillofacial Radiology, School of Dentistry, Shahrekord University of Medical Sciences, Shahrekord, Iran; ^5^ Department of Oral and School of Maxillofacial Surgery, School of Dentistry, Shiraz University of Medical Sciences, Shiraz, Iran; ^6^ Department of Orthodontics, Faculty of Dentistry, Tehran University of Medical Sciences, Tehran, Iran; ^7^ Department of Orthodontics, Faculty of Dentistry, Tabriz University of Medical Sciences, Tabriz, Iran

**Keywords:** Periodontitis, Volatile Organic Compounds, Hydrogen Sulfide, Methanethiol

## Abstract

Periodontitis is a notable public health issue impacting more than 1 billion individuals globally, and its connection with volatile organic compounds (VOCs) has attracted growing interest. This review seeks to explore the existing knowledge on the link between VOCs and periodontitis. Materials and Methods: An extensive literature review was performed to pinpoint key VOCs associated with periodontitis. Results: The review revealed that several VOCs, such as hydrogen sulfide, methanethiol, indole, limonene, formaldehyde, 1,4-dichlorobenzene, 2-Aminothiazoline-4-carboxylic acid, ethyl acetate, methyl mercaptan, dimethyl sulfide, acetone, pyridine, picolines, o-xylene, mandelic acid, and N-acetyl-S-(4-hydroxy-2-butenyl)-L-cysteine, are linked to periodontitis, with some contributing to heightened oral infection, direct tissue harm, oral malodor, and inflammatory responses, while the causality of this phenomenon remains unclear as it is uncertain which event occurs first. Conclusion: This review enumerates the VOCs that may either contribute to or arise from periodontitis; hydrogen sulfide appears to be the most extensively studied VOC in the context of periodontitis. This review highlights the intricate relationship between VOCs and periodontitis and underscores the necessity for additional research to clarify the mechanisms underlying this association and to guide the creation of effective prevention and treatment strategies.

## Introduction

The concept of VOCs encompasses a broad range of chemical substances characterized by their carbon-based composition and volatility at ambient temperatures [1]. These compounds can be categorized into distinct families based on their chemical formulas, each exhibiting unique properties and varying levels of toxicity [1]. However, the definition and classification of VOCs are not universally agreed upon, with different countries and regulatory frameworks employing distinct criteria and methodologies to determine VOC content and volatility [2][3]. Furthermore, the term "volatile organic compound" is often poorly defined, leading to inconsistencies in standardized tests and regulations [4]. According to Võ and Morris [5], very volatile organic compounds (VVOCs) are a subgroup of indoor pollutants that lack a clear and internationally accepted definition, despite being recognized by the World Health Organization (WHO).

The pervasive presence of VOCs in both indoor and outdoor environments has sparked considerable concern due to their potential adverse effects on human health [6]. Research has shown that exposure to VOCs can have detrimental consequences, including contributing to the development of asthma and allergy in children and adults [7][8]. The analysis of urinary VOC metabolites has provided valuable insights into the effects of VOC exposure on human health, with findings indicating a correlation between VOC exposure and reduced lung function in adults [9]. Additionally, the calculation of an environment risk score and amounts of exposure to VOCs can be used to partially predict mortality in certain cases [10].

The prevalence of periodontitis worldwide is a significant concern, with more than one thousand million individuals impacted by serious gum disease in two thousand and twenty-one, leading to a worldwide age-standardized incidence of twelve point five zero percent [11]. This condition is projected to increase by 44.32% by 2050, affecting more than 1.5 billion people [11].

The ambiguity surrounding the risk factors of periodontitis has been a longstanding concern in the field of periodontology, with various studies attempting to elucidate the underlying mechanisms and associations. The complexity of periodontitis risk factors is further compounded by the existence of multiple factors, including personal behaviors and professional practices, which can influence disease progression and prognosis. Therefore, it is essential to develop multifactorial models that can accurately assess risk and inform prevention and intervention strategies. despite the established links between VOC exposure and various health issues, including respiratory problems and sleep disorders, the relationship between VOCs and periodontitis remains poorly understood; we aimed at investigating the association between VOCs and periodontitis. Also, what makes this study novel is its attempt to bridge the knowledge gap by exploring the potential link between VOC exposure and periodontitis, which has not been thoroughly examined in the existing literature, and by developing a multifactorial model that considers the complex interplay of risk factors contributing to periodontitis, thereby providing new insights into the prevention and intervention strategies for this debilitating condition.Top of Form

## potential health risks of different categories of volatile organic compounds

VOCs can be broadly categorized into several distinct groups, including alkanes, alkenes, alkynes, aromatics, and halogenated compounds, each with its own unique characteristics and potential health risks [12]. According to Phillips et al., alkanes and benzene derivatives have been found in the breath of patients with lung cancer, suggesting their potential use as biomarkers for the disease [13]. Similarly, Besis et al. reported that alkanes were the dominant VOCs in vehicular cabin air [14]. Furthermore, Wickliffe et al. [15] and Phillips et al. [16] found that alkanes were associated with oral malodor and oxidative stress, as well as increased long-term health risks in residential indoor air, respectively. The correlation between alkanes and other VOCs, such as BTEX compounds, was also noted in [16]. Research has shown that benzene exposure can modulate signaling pathways involved in the modulation of cellular reaction to oxidative stress, which can lead to cell proliferation and apoptosis [17].

Alkenes, a type of VOC, have been found to contribute significantly to the total VOC concentration in different environments. For instance, in a regional background site in China, alkenes contributed 10.3% to the total VOC concentration [18]. Similarly, in a petrochemical area, alkenes and oxygenated volatile organic compounds (OVOCs) exhibited higher ozone formation potential (OFP) [19]. In addition, a study on vehicular cabin air found that alkenes were among the dominant VOCs present, with a significant contribution to the total VOC concentration [14].

Alkynes, a class of unsaturated hydrocarbons, are known to be significant contributors to the ozone forming potential (OFPs) of VOCs. According to a study on the characteristics of VOCs emitted from biofuel combustion in China, alkenes and alkynes were found to have the highest OFPs values, with aldehydes accounting for over 50% of the total OFPs [20]. An additional investigation into the traits and source allocation of VOCs in the automotive industrial area of Shanghai indicated that alkynes comprised 6.8% of the overall VOCs level, with alkanes, alkenes, aromatics, and halogenated hydrocarbons being the other principal constituents [21]. The atmospheric chemical responsiveness was gauged using the peak incremental reactivity (MIR) and hydroxyl radical depletion rate, which demonstrated that alkynes had a crucial impact on ozone creation [21]. A study on the traits and source distribution of VOCs in the northern outskirts of Nanjing found that alkynes accounted for 7.3% of the total VOCs mixing ratios, with a significant seasonal variation in their concentrations [22]. The study also reported that the observation site was greatly affected by the surrounding industrial areas, with alkynes being one of the major VOCs species [22]. Another study on the characteristics and sources of VOCs at different ozone concentration levels in Tianjin found that alkynes accounted for 2.73%-4.13% of the VOCs concentrations, with a higher proportion during periods of excellent ozone concentration [23]. The study also reported that the main VOCs species included propane, ethane, ethylene, toluene, and acetylene, among others [23].

Aromatic VOCs have been found to have significant effects on human health and the environment [24][25][26][27]. Toluene, benzene, and styrene, which are widely distributed in the environment, have been shown to induce inflammatory reactions in lung cells and increase the expression of COX-2 and prostaglandins [24]. Additionally, exposure to benzene, ethylbenzene, and xylene (BEX) has been linked to hearing loss in adults [25]. The relationship between VOCs and health effects is complex, and further research is needed to fully understand the mechanisms by which these compounds affect human health [25][26]. VOCs are also significant pollutants in the petrochemical industry, and controlling aromatic hydrocarbons is crucial for managing VOCs [26].

The presence of halogenated VOCs has been detected in various environments, including indoor air [27][28], seawater [29], and bottled mineral water [30]. These compounds, which include chloroform and carbon tetrachloride, are formed through the reaction of sodium hypochlorite with organic chemicals in household cleaning products [28]. The use of such products has been shown to significantly increase the concentrations of halogenated VOCs in indoor air [28]. Additionally, halogenated VOCs have been found to be naturally produced by marine macroalgae [29] and have been detected in fish samples from polluted areas [31]. The analysis of these compounds is crucial due to their potential harmful effects on human health and the environment [32]. Various methods have been developed for the analysis of halogenated VOCs, including headspace chromatography [30] and gas-chromatographic determination [31].

## VOCs, infection, and periodontitis

Studies have demonstrated that disease-causing infections triggered by microorganisms like Porphyromonas gingivalis, Treponema denticola, and Tannerella forsythia can result in the generation of volatile sulfur substances (VSS) and other harmful substances, leading to bad breathing smell and gum diseases [33].

Research has shown that certain oral bacteria, such as Porphyromonas gingivalis, Prevotella intermedia, and Streptococcus mutans, produce unique VOC profiles that can be used to identify and distinguish between different bacterial species [33][34][35][36]. These VOCs include compounds such as hydrogen sulfide, methanethiol, and indole, which are produced through various metabolic processes [33][34]. Furthermore, the analysis of VOCs in saliva has been proposed as a potential diagnostic tool for oral cancer and other diseases [35][36]. Furthermore, studies have also investigated the identification of fragrant subgingival and tongue microorganisms in individuals with diabetes and those without diabetes who have oral halitosis, highlighting the importance of oral hygiene practices such as interdental flossing in reducing the likelihood of oral malodour [33][34][35][36]. Research has shown that certain oral pathogens, such as Porphyromonas gingivalis, can produce a variety of VOCs, including hydrogen sulphide, methanethiol, acetone, and dimethylsulphide, which can be used as markers for bacterial cell growth and response to treatment [37].

Additionally, the use of egg yolk immunoglobulin (IgY) has been found to inhibit the growth of P. gingivalis and reduce the production of VOCs and VSCs [38]. Microbial metabolism can be elucidated through VOC analysis, revealing nuances in bacterial responses to stressors. Milanowski et al. study’s findings underscore the importance of considering strain-specific variations in VOC profiles, as well as the impact of subtle concentration changes on bacterial physiology. Elucidation of these dynamics can inform the development of novel therapeutic strategies, leveraging the antimicrobial properties of silver ions to mitigate disease progression [39].

The aetiopathogenesis of halitosis, another term for oral malodour, involves the production of VSCs by oral microorganisms, particularly gram-negative anaerobic bacteria [40][41]. VSCs, such as hydrogen sulphide, methyl mercaptan, and dimethyl sulphide, are produced by the bacterial breakdown of proteinaceous substrates in the oral cavity and are considered the primary cause of oral malodour [42][43]. The relationship between oral malodour and VSC-producing bacteria has been investigated, with studies suggesting that these bacteria, particularly those colonizing the lingual dorsum, play a significant role in the generation of halitosis [42][44]. Furthermore, research has shown that oral malodour is often associated with periodontal diseases and tongue coating, with significant correlations observed between VSC values and periodontal conditions [43][44]. The management of oral malodour typically involves the use of mouthwashes and other oral hygiene measures, with some studies suggesting that zinc salts may be effective in inhibiting VSC formation [42][43][44].

## VOCs, inflammatory pathways, and Periodontal Disease

A study examining the effects of formaldehyde, as a VOC, on woodworkers found that long-term exposure to formaldehyde resulted in a statistically significant worsening of periodontal tissue condition [45]. Furthermore, an inquiry into the impacts of formaldehyde on vascular endothelial growth factor, matrix metalloproteinase 2, and osteonectin concentrations in periodontal membrane and alveolar bone in rodents disclosed that formaldehyde poisoning can disturb the periodontal membrane and induce collagen fiber deterioration [46]. Additionally, a study on the pulpal changes associated with advanced periodontal disease found that pulpal calcification and partial necrosis of pulp were common findings in teeth affected by severe periodontitis, which may be related to the use of formalin in storing the teeth [47]. Research has shown that VOCs, including alkanes, are present in the breath of individuals with oral malodor, which is often associated with periodontal disease [16]. These VOCs are produced as a result of oxidative stress, which is characterized by the peroxidation of polyunsaturated fatty acids in cell membranes [16]. But VOCs are containing wide range of chemical that some might even decrease the risk of periodontitis. Limonene, a compound found in lemon essential oil, has been investigated for its potential effects on oral health, including its influence on the progress of early caries [48]. While the primary focus of these studies has not been periodontitis, the antimicrobial and anti-inflammatory properties of limonene suggest it may have a beneficial impact on periodontal health [49][50]. For instance, essential oils containing limonene have been studied for their anti-inflammatory potential in the context of supportive periodontal therapy, indicating a possible role in managing periodontitis [50]. However, more direct research is needed to fully understand the effects of limonene on periodontitis. Further studies are required to elucidate the specific relationship between limonene and periodontitis, considering the complex interplay of factors involved in periodontal disease [51].

## Real world evidence

**Table:** Table1. summary of VOCs of interest captured from reviewed literature

**VOC**	**Summary**
Hydrogen sulfide [33][42]	Causes bad breath and disease
Methanethiol [33][34]	Contributes to oral malodor formation
Indole [33][34]	Associated with oral cancer diagnosis
Limonene [48][49]	May reduce oral infection risk
Formaldehyde [45][46]	Damages periodontal tissue and bone
1,4-dichlorobenzene [59]	Increases periodontitis risk significantly found
2-Aminothiazoline-4-carboxylic acid [60]	Mediates VOC exposure and periodontitis
Ethyl acetate [58]	Correlated with bacterial communities found
Methyl mercaptan [42][63]	Contributes to halitosis and disease
Dimethyl sulphide [33][42]	Associated with oral malodor and
Acetone [37]	Produced by oral pathogens bacteria
Pyridine [52]	Elevated in periodontitis patients saliva
Picolines [52]	Found in periodontitis patients saliva
o-xylene [59]	Associated with periodontitis in adults
Mandelic acid [61]	Positively associated with periodontitis risk
N-acetyl-S-(4-hydroxy-2-butenyl)-L-cysteine [61]	Contributed to periodontitis risk significantly

The relationship between periodontitis and VOCs has been a subject of interest in recent studies as mentioned in Table-1.

Kostelc et al. in 1981 for first time investigated the presence and quantification of volatile aromatic amines, specifically pyridine and picolines, in the saliva of individuals with healthy mouths versus those with periodontitis. Utilizing gas chromatography/mass spectrometry, researchers found these compounds were nearly absent in healthy subjects but significantly elevated in those with periodontitis, reaching 636.4 ng/5 mL of saliva (SEM 154.7). The findings suggest these volatiles could play a role in the disease’s etiology or serve as potential diagnostic markers [52]. Later in 1984, Utilizing gas chromatography, Kostelc et al. analyzed volatile compounds in mouth air and saliva of participants undergoing a controlled gingivitis experiment. Notably, sulfurous compounds intensified in conjunction with gingivitis onset, while salivary volatile production fluctuated in response to periodontal health [53].

Research has shown that individuals with periodontal disease tend to have higher concentrations of VSCs in their mouth air, which can lead to halitosis [53]. A study conducted by Vandekerckhove et al. (2009) [54] found that there is a significant correlation between the concentration of VSCs and the severity of periodontal disease.

Research has shown that VOCs are present in the breath of patients with oral squamous cell carcinoma and can be used as a signature for the disease [55]. The levels of VSCs in periodontal pockets have been found to be associated with the severity of periodontitis and can impact the outcome of initial periodontal therapy [56]. Furthermore, research has shown that there is a significant correlation between VSCs and periodontal parameters, such as periodontal probing depth and pocket depth [57]. Additionally, studies have found that periodontal therapy can lead to a significant decrease in oral malodour and periodontal parameters [56][57].

Multiple investigations have examined the connection between VOCs and periodontal disease, emphasizing the substantial influence of particular VOCs on periodontal well-being. One study by Liang et al. (2024) [58] explored the odorous VOCs released from bio-decomposition and their interaction mechanisms with bacteria. The research highlighted that volatile sulfur-containing compounds (VOSCs) and oxygenated volatile organic compounds (OVOCs) were the primary emissions from food waste fermentation, contributing to unpleasant odors. The study also noted that intra-type and inter-type food waste harbored similar yet distinct bacterial communities, with ethyl acetate, 2-butanone, and VOSCs showing correlations with these microbial communities. Key pathogens identified included Enteroccus, Proteobacteria, Mycobacterium, and Salmonella.

Dong et al. (2024) [59] discovered that a consistent twofold rise in 1,4-dichlorobenzene was linked to a 16% rise in the likelihood of having periodontal disease. The weighted quantile sum (WQS) regression model disclosed that 1,4-dichlorobenzene was the most crucial contributor to the association between VOC co-exposure and periodontal disease, with totalbilirin levels mediating this association by 48.32%. Dai et al. (2024) [60] examined the mediating function of systemic inflammation markers, such as leukocyte and lymphocyte counts, in the relationship between VOC exposure and periodontal disease. Utilizing NHANES 2011-2014 data, they discovered that systemic inflammation partially mediated the association between VOC exposure and periodontal disease. Bayesian kernel machine regression (BKMR) and quantile g-computation (QGC) models confirmed that mixed VOC exposure was significantly linked with periodontal disease, with 2-Aminothiazoline-4-carboxylic acid (ATCA) being the most influential. Jiang et al. (2024) [61] further explored the mediating function of immune cells and found that urinary levels of 2-aminothiazoline-4-carboxylic acid, mandelic acid, and N-acetyl-S-(4-hydroxy-2-butenyl)-L-cysteine were positively linked with periodontal disease risk. WQS models indicated a positive correlation between VOC mixtures and periodontal disease risk, with 2-amino thiazoline-4-car boxylic acid being the most crucial contributor, and monocytes playing a vital role in the observed association. Silva et al. (2024) [62] provided insights into the connection between VSCs, biofilm, and periodontal well-being. The study, involving 64 patients with periodontal disease and 60 periodontally healthy individuals, found that a notable tongue coating index and a decreased tooth count were significantly higher in the periodontal disease group. Unstimulated salivary flow rate below 0.25 mL/min was also statistically significant in the periodontal disease group (p = 0.032). Lee et al. [63] investigated VSC levels and halitosis in patients with gingivitis and periodontal disease, finding that VSC levels were significantly higher in both gingivitis and periodontal disease groups compared to healthy controls. Halitosis was more prevalent in gingivitis (39.5%) and periodontal disease (42.9%) patients than in healthy controls (3%). Bivariate logistic regression showed that periodontal disease significantly increased the likelihood of halitosis by 3.607 times. Bolepalli et al. [64][65] found that 80% of participants showed varying degrees of halitosis when assessed organoleptically, and 74.6% when measured with a Halimeter. The study evaluated 240 subjects, including 60 without periodontal disease and 180 with gingivitis and periodontal disease, and found that key parameters like plaque index, gingival index, and periodontal depth were significantly linked with oral malodor (P < 0.001). Xue et al. (2024) [66] investigated the association between blood ethylene oxide levels and the prevalence of periodontal disease, finding that individuals in the periodontal disease group had significantly higher HbEO levels (40.57 vs. 28.87 pmol/g Hb, p < 0.001). The highest Hb EO quartile exhibited an increased risk of periodontal disease (OR = 2.88). Makino et al. [67] explored the link between VSCs and the progression of periodontal disease in elderly non-smokers, finding that those in the highest V SC group had a 33% higher rate of periodontal disease progression compared to the lowest V SC group, after adjusting for factors like sex, number of remaining teeth, and initial periodontal status.

## Conclusion

Reviewed literature in this study shows that research have consistently shown that periodontitis exhibits higher concentrations of VSCs, which are produced by periodontal bacteria and can contribute to the development of halitosis. The presence of VSCs, such as hydrogen sulfide and methyl mercaptan, has been linked with the magnitude of periodontal parameters with a positive correlation, and their levels have been found to decrease significantly following periodontal therapy. Furthermore, research has identified a significant association between VSCs and periodontal parameters, including probing depth, clinical attachment level, and bleeding on probing. The role of systemic inflammation and immune cells, such as monocytes, has also been implicated in the connection between VSCs and periodontal ailment, with studies suggesting that these factors may mediate the association between VSC exposure and disease progression.

## Conflict of Interest

The authors have no conflicts of interest relevant to this article to disclose.
